# Modern Economy in Sanatorium Management

**Published:** 1913-09-13

**Authors:** 


### The Hospital

The Workers' Newspaper of Administrative Medicine and Institutional Life, Administration, National Insurance and Health.

No. 1420, Vol. LIV. SATURDAY, SEPTEMBER 13, 1913. PRICE ONE PENNY

### MODERN ECONOMY IN SANATORIUM MANAGEMENT.

The first reports on the working of what has come to be known as "sanatorium benefit" are now beginning to come in. Pride of place must be given to the record of the Welsh National Memorial to King Edward VII. The Council of this movement, calculating that of the one and a half million sterling made available under the National Insurance Act for the provision of sanatoria Wales and Monmouth could claim £81,000, announce their intention of making this sum up to £135,000, which is the estimated total capital cost of their scheme for Wales. These are brave words, but the work already accomplished makes them sound feasible. "In July 1912 the West Wales Sanatorium was the only institution controlled by the Association. The accommodation there was for twenty-four patients. On March 31 last the Association had approximately 200 beds at their disposal in four sanatoria," while arrangements have been made in addition for the accommodation of Welsh cases at six English sanatoria. This is pending the erection of two large home institutions for North and South Wales containing in all 404 adult and 80 children's beds. As to hospital beds for advanced cases, 178 are secured, and it is stated that in a very short time there will be available a further 184. The Council estimates that during the year ending March 1914 the complete scheme, so far as that relates to hospitals, will be in full working order.

The only point to escape due attention, it seems to us, is that of "after-care." There is no doubt that something more than exhorting patients on their discharge and making representations to their employers is needed in this matter. The leaflets to this effect rather tentatively issued by the National Association for the Prevention of Tuberculosis, through the secretaryship of Dr. McConnel, seem to us to throw upon the sanatorium resident duties which appropriately belong to the tuberculosis officer. But the sanatorium resident, as may be seen at Dr. McConnel's own institution at Kelling, has a very real duty, too, in the direction of after-care, and probably a much more important one. As was pointed out in a series of articles published in these columns on "Institution Outdoor Work and Administration," there is at each sanatorium a good deal of commissariat and other work that can be undertaken by ex-patients—market gardening, pig and poultry raising, the work of a small holding and even that of a farm, and also horse-keeping, station work, and coal leading. The fact that at Frimley, whence "graduated labour" arose, the grounds are of small extent, has made people think that consumptives are only to be employed in digging foundations, or work of that description. But whereas there is a limit to requirements in this direction, there is none in the direction of marketing vegetable and animal produce. The real model for modern economic sanatorium management is the large public mental hospital. However, the Welsh scheme as begun reflects great credit upon the philanthropic and energetic employers of that fine organiser, Dr. Paterson.

But combination over an area the size of Wales can, of course, happen very seldom; and smaller authorities who are, so to say, "playing a lone hand," must do as best they can. Nor, too, has every English county behind it a rateable value anything like that of Yorkshire or Lancashire. Dr. Moss-Blundell, County Medical Officer of Health for Huntingdon, has issued an interesting "first annual report on the administration of sanatorium benefit," which represents much better what the ordinary insuree may expect than does the early established, thorough-going scheme just mentioned. There were in all sixty-one applications for sanatorium benefit, less than one-third of whom received institutional treatment. It is remarkable that in Huntingdon the rural districts show more tuberculosis than the urban ones, and this Dr. Moss-Blundell thinks may be concerned somewhat with the different housing conditions in the country; at all events, in the case of female patients. The appointment of a county tuberculosis officer has, from what one can gather, not been yet made, and the Medical Officer of Health himself has been doing the work. This is not a very good plan as a permanency, although perhaps as satisfactory as the employment of one or two recently successful candidates for these posts. Neighbouring counties have combined before now in order to supply themselves with sanatorium accommodation, but where the work is largely domiciliary this would not be possible. However, nearly every county should be able to support one tuberculosis officer. In the case of very small areas, tuberculosis work might be combined with a school appointment; that is, for candidates possessing special knowledge in both directions, and such posts would suit well those who value the work chiefly as a stepping-stone to public health employment. In the larger centres there ought, at any rate, to be found rather the special tuberculosis clinician and sanatorian; and in time, perhaps, the phthisiological specialist. Nothing is more instructive than this comparison of a small county's experience with that of a large area like Wales.

